# Supplementary material for: Evaluation of rural comprehensive development level and obstacle factors in various countries around the world
Source: PLoS One. 2025 Apr 18;20(4):e0317282. doi: 10.1371/journal.pone.0317282 (PMC12007719; doi:10.1371/journal.pone.0317282)
Supplement: Attachment — Submitted filename: renamed_a50a1.docx [file pone.0317282.s001.docx]

1、 The abstract appears to have missing parts and the research results are not represented
2、 The introduction seems to be thorough. No comments.
3、 I suggest to add a theoretical framework on the level of integrated rural development to better reflect the theoretical value.
4、 The title data sources and research methods, It seems to have been disordered, suggesting that the methodology be brought forward
5、 Is AHP validated? What are the results of the consistency validation?
6、 How is the comprehensive weight of the indicator system calculated? (Table 3) What are the weights of the two parts (AHP, entropy weight)? Need to be reflected in the article.
7、 Please provide more details regarding the input data used throughout this research. Some supporting references would be helpful for the future readers to justify the data selection.
8、 At the results and analysis section, i suggest to draw figures of spatial pattern in three dimensions（agricultural development, rural development, farmers’ lives）, in addition to the Figure 2.
9、 At the same time, the paper needs to focus on analyzing differences in spatial patterns about 175 countries and regions.
10、 The conclusions section should expand on limitations of this study and future research needs. I suggest listing the bullet points.
11、 The recommendations section could be written separately.
