# Supplementary material for: Evaluation of rural comprehensive development level and obstacle factors in various countries around the world
Source: PLoS One. 2025 Apr 18;20(4):e0317282. doi: 10.1371/journal.pone.0317282 (PMC12007719; doi:10.1371/journal.pone.0317282)
Supplement: Attachment — Submitted filename: Email clarification.docx [file pone.0317282.s003.docx]

**To the Editor and Anonymous Reviewers:**

The authors express their sincere gratitude for the feedback provided in the email, and we particularly value the opportunity given by the journal to refine our manuscript. Below, we detail the modifications made in response to the comments made in the email.

1.It appears that your ORCiD iD has not been validated in your Editorial Manager account and we are unable to proceed until that step is complete.

I have already added my ORCiD iD in the system.

2.Please ensure that you include a title page within your main document. You should list all authors and all affiliations as per our author instructions and clearly indicate the corresponding author.

I have listed all authors and their affiliations according to the author instructions and clearly indicated the corresponding author.

3. We note that Figures 2 in your submission contain [map/satellite] images which may be copyrighted. All PLOS content is published under the Creative Commons Attribution License (CC BY 4.0), which means that the manuscript, images, and Supporting Information files will be freely available online, and any third party is permitted to access, download, copy, distribute, and use these materials in any way, even commercially, with proper attribution. For these reasons, we cannot publish previously copyrighted maps or satellite images created using proprietary data, such as Google software (Google Maps, Street View, and Earth). For more information, see our copyright guidelines: http://journals.plos.org/plosone/s/licenses-and-copyright.

I have replaced the copyright information for Figure 2 and updated the title of Figure 2 with the source information (Fig.2. World map depicting the distribution of rural comprehensive development level,Based on the public domain imagery from Natural Earth ([http://www.naturalearthdata.com](http://www.naturalearthdata.com/)), modified for illustrative purposes.).

4.Please state what role the funders took in the study. If the funders had no role, please state: "The funders had no role in study design, data collection and analysis, decision to publish, or preparation of the manuscript."

I have already stated it in the Cover Letter.
